# Risk Factors for Lead-Related Venous Obstruction: A Study of 2909 Candidates for Lead Extraction

**DOI:** 10.3390/jcm10215158

**Published:** 2021-11-03

**Authors:** Marek Czajkowski, Wojciech Jacheć, Anna Polewczyk, Jarosław Kosior, Dorota Nowosielecka, Łukasz Tułecki, Paweł Stefańczyk, Andrzej Kutarski

**Affiliations:** 1 Department of Cardiac Surgery, Medical University of Lublin, 20-090 Lublin, Poland; mczajkowski@interia.pl; 2 2nd Department of Cardiology, Faculty of Medical Science in Zabrze, Medical University of Silesia in Katowice, 41-800 Zabrze, Poland; wjachec@interia.pl; 3 Department of Physiology, Patophysiology and Clinical Immunology, Collegium Medicum of Jan Kochanowski University, 25-317 Kielce, Poland; 4 Department of Cardiac Surgery, Świętokrzyskie Center of Cardiology, 25-736 Kielce, Poland; 5 Department of Cardiology, Masovian Specialistic Hospital of Radom, 26-617 Radom, Poland; jaroslaw.kosior@icloud.com; 6 Department of Cardiology, The Pope John Paul II Province Hospital of Zamość, 22-400 Zamość, Poland; dornowos@wp.pl (D.N.); paolost@interia.pl (P.S.); 7 Department of Cardiac Surgery, The Pope John Paul II Province Hospital of Zamość, 22-400 Zamość, Poland; luke27@poczta.onet.pl; 8 Department of Cardiology, Medical University of Lublin, 20-090 Lublin, Poland; a_kutarski@yahoo.com

**Keywords:** lead-related venous obstruction, transvenous lead extraction, risk factors for venous obstruction, abandoned lead

## Abstract

Background: our knowledge of lead-related venous stenosis/occlusion (LRVSO) remains limited and there is still controversy regarding the risk factors for LRVSO. Venography is mandatory before transvenous lead extraction (TLE). Methods: we performed a retrospective analysis of venograms in 2909 patients (39.43% females, average age 66.90 years) who underwent TLE between 2008 and 2021 at high-volume centers. Results: the severity of LRVSO was likely to be dependent on the number of leads in the system (OR = 1.345; *p* = 0.003), the number of abandoned leads (OR = 1.965; *p* < 0.001), the presence of coronary sinus leads (OR = 1.184; *p* = 0.056), male gender (OR = 1.349; *p* = 0.003) and patient age at first CIED implantation (OR = 1.008; *p* = 0.021). The presence of permanent atrial fibrillation (OR = 0.666; *p* < 0.001) and right ventricular diastolic diameter (OR = 0.978; *p* = 0.006) showed an inverse correlation with the degree of LRVSO. The combined three-model multivariate analysis provided better prediction of LRSVO using the above-mentioned factors than the CHA2DS2-VASc score. Conclusions: the severity of LRVSO is probably dependent on the mechanical impact of the implanted/abandoned leads on the vein wall, therefore the study has demonstrated the central role of system-/procedure-related risk factors. The thrombotic mechanism may be less important, especially long after implantation, and for this reason the combined prediction model for LRVSO in this study was more effective than the CHA2DS2-VASc score.

## 1. Introduction

Venous obstruction following transvenous pacemaker implantation has frequently been described in the literature [1,2,3,4,5,6,7,8,9,10,11,12,13,14,15,16,17,18,19,20,21,22]. Despite over 55 years of experience with transcutaneous cardiac pacing and ample evidence, the risk factors that contribute to venous stenosis have not been clearly identified (confirmed in independent studies) [23]. Lead-related venous obstruction (stenosis/occlusion) (LRVSO) usually remains asymptomatic (except acute/early axillary vein thrombosis) but makes it difficult or even impossible to implant a new lead or to insert port-a-cath and hemodialysis catheters [1,2,3,4,5,6,7,8,9,10,11,12,13,14,15,16,17,18,19,20,21,22,23,24,25,26]. The available studies on risk factors for LRVSO have conflicting results [1,2,3,4,5,6,7,9,10,11,13,14,15,16,17,18,19,20], but it is still reasonable to expect that the identification of modifiable patient-related, system-/lead-related and lead management-related risk factors can help reduce the incidence and severity of LRVSO.

Transvenous lead extraction (TLE) is an essential method of managing lead-related problems [27,28,29], and preoperative venography facilitates the choice of the most suitable TLE strategy. Thus, according 2017 HRS and 2018 EHRA experts’ consensus statement on lead extraction [28,29] venography is recommended and helpful before lead extraction procedures in patients without contraindications for contrast administration. Out of the 22 reports on LRVSO, only two studies were carried out in populations over 200 patients [1,2], 10 with 100–150 participants [3,4,5,6,7,8,9,10,11,12] and the remaining 10 studies in populations consisting of 30–89 individuals [13,14,15,16,17,18,19,20,21,22]. Knowledge of LRVSO has been gained from studies in 2299 patients, but the risk factors have been assessed only in 2012 patients and reported in 18 papers [1,2,3,4,5,6,7,9,10,11,13,14,15,16,17,18,19,20]. We decided to perform a detailed analysis of risk factors for LRVSO in 2909 patients treated by the same very experienced operator at three high-volume centers.

The aim of this study was to determine patient-, system-/lead-, history of pacing-, and previous lead management-related risk factors for LRVSO, including the influence of anticoagulation or antiplatelet treatment on venous obstruction.

## 2. Material and Methods

### 2.1. Study Population

This post-hoc analysis used clinical data of 2909 patients who underwent transvenous lead extraction (TLE) performed by one experienced operator between June 2008 and March 2021 at three high-volume centers. 

Information about patient history of pacing such as: presence of abandoned leads before TLE, number of abandoned leads before TLE, presence of multiple abandoned leads before TLE, presence of more than 4 and 5 leads in heart before TLE, presence of two dual-coil ICD leads before TLE, three ICD leads before TLE, presence of leads on both sides of the chest before TLE, previous TLE before present TLE, history of early CIED intervention, upgrading or additional lead implantation, upgrading or downgrading with lead abandonment, dwell time of the oldest lead per patient before TLE and cumulative dwell time of extracted leads before TLE were entered into a computer database and then analyzed and compared with different degree of venous obstruction. 

Patients with medical contraindications for venography (contrast intake) were excluded from the study.

### 2.2. Venography

Preoperative venography was performed in 2909 patients who were submitted for transvenous lead extraction. An intravenous catheter was placed in the peripheral arm vein on the side or sides of endocardial lead implantation. All patients received an injection of 20–40 mL high-quality contrast medium (350 mg iodine/mL) and venous blood flow in the upper arm, neck and chest was recorded by cine-angiography. All images were acquired in the anteroposterior view. 

An experienced cardiologist and experienced (trained by an interventional radiologist) cardiac surgeon reviewed the venograms, and venous patency was graded on a 5-degree scale from normal flow to complete occlusion. All venograms were obtained in the same manner. 

**Figure 1:**
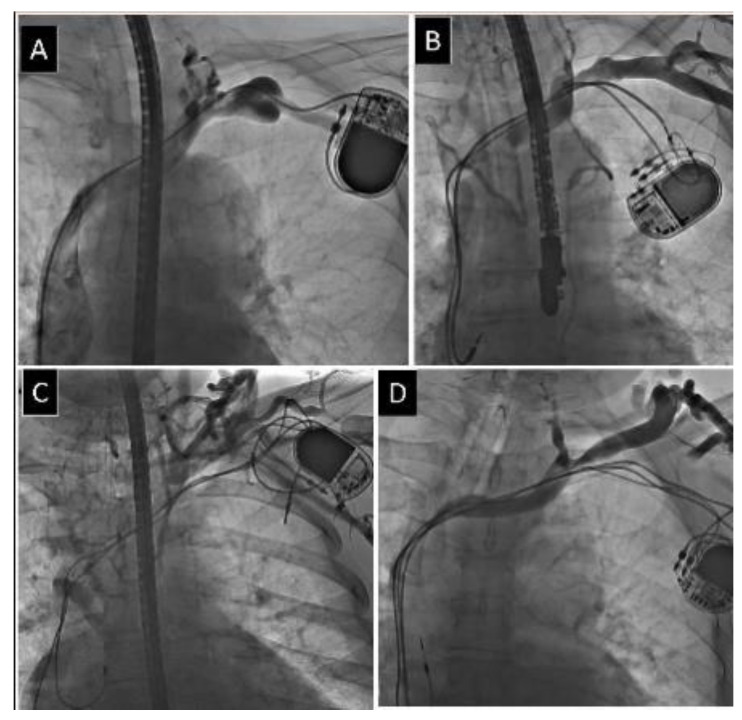
Different degree of obstruction of axillary vein. (**A**) Mild narrowing of axillary vein. (**B**) Moderate narrowing of axillary vein. (**C**) Severe narrowing of axillary vein. (**D**) Complete occlusion of axillary vein.

**Figure 2:**
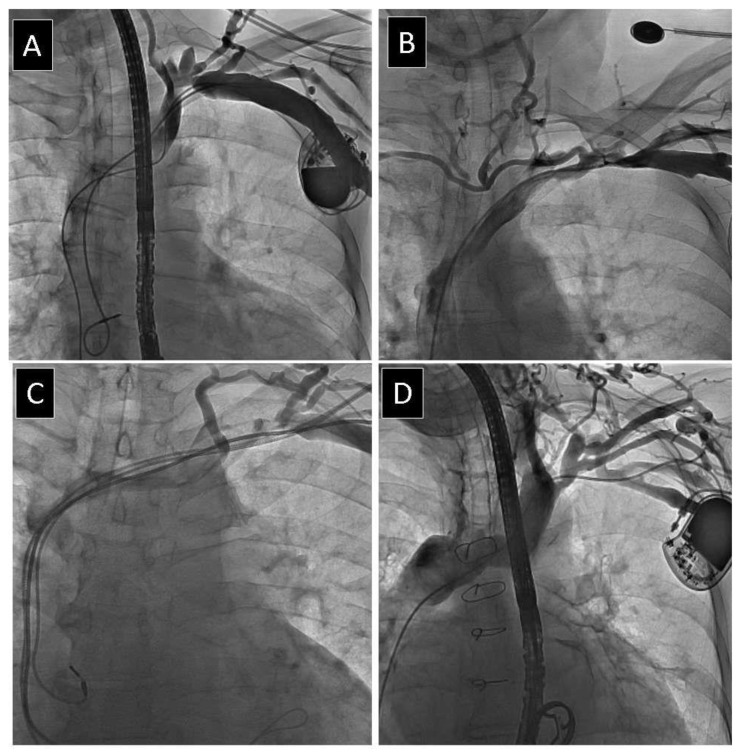
Different degree of obstruction of subclavian vein. (**A**) Mild narrowing of subclavian vein. (**B**) Moderate narrowing of subclavian vein. (**C**) Severe narrowing of subclavian vein. (**D**) Complete occlusion of subclavian vein.

**Figure 3:**
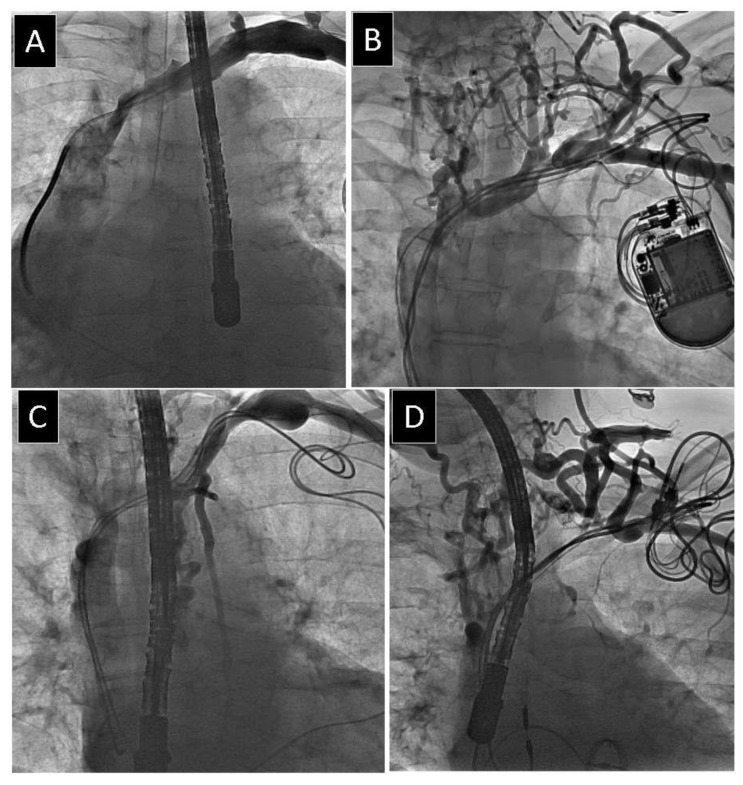
Different degree of obstruction of anonymous vein. (**A**) Mild narrowing of anonymous vein. (**B**) Moderate narrowing of anonymous vein. (**C**) Severe narrowing of anonymous vein. (**D**) Complete occlusion of anonymous vein.

The narrowest and widest points of the target vessel for lead placement were identified by visual inspection to obtain minimum and maximum venous diameters, and measurements from two to three individually calibrated frames were averaged to determine the final status of the vein as no stenosis, mild stenosis (<1/3 reduction in venous lumen), moderate stenosis (1/3 to 2/3 reduction in venous lumen), severe stenosis (≥2/3 reduction in venous lumen, but still patent) and complete occlusion of the axillary (AxV) (Figure 1) subclavian (ScV) (Figure 2) innominate (brachiocephalic) (AnV) (Figure 3) veins and superior vena cava (SVC). 

Contrast injection on the lead side, thanks to collateral circulation through the neck and thoracic wall veins, enabled the contralateral brachiocephalic vein patency rating in some patients. 

Dynamic venography showing the total venous flow allows for approximate, subjective estimation of conditions for future AV fistula patency in case of lead total removal. Arterio-venous fistula should not be located on the leads’ side if any leads remain or if severe stenosis or occlusion resulting in slow venous flow is found.

What is the practical significance of this classification of vessel narrowing for the cardiologist, anesthesiologist or surgeon?

Mild narrowing: possible insertion of a new/additional lead using standard introducers, central venous catheters, permanent catheters for hemodialysis, with a chance that arteriovenous (AV) fistula for hemodialysis access will work properly.

Moderate narrowing: probable insertion of a new lead but hydrophilic guide wires and longer introducers are necessary, possible insertion of central venous catheters (troubles possible), possible insertion of permanent catheters for hemodialysis and there is a small chance that AV fistula for hemodialysis access will work properly. Fistula patency is evaluated on the basis of collateral flow, if present.

Severe narrowing: impossible insertion of a new lead, hydrophilic guide wires and longer introducers might be helpful, insertion of central venous catheters may be risky, chances to pass a catheter for hemodialysis without venoplasty are very low or nonexistent, and there is usually a small chance that AV fistula for hemodialysis access will work properly. Fistula patency is evaluated on the basis of collateral flow, if present.

Complete occlusion: no chance to pass a hydrophilic guide wire; only lead extraction and regaining venous access enables insertion of a new lead. Fistula patency is evaluated on the basis of collateral flow, if present.

Reuse of occluded veins and technical aspects of lead extraction/replacement depend not only on maximal venous narrowing but also on the length of the narrowing (the number of the affected vessels, too).

### 2.3. Lead Extraction Procedure

Lead extraction procedures were defined according to the most recent guidelines on the management of lead-related complications (HRS 2017 and EHRA 2018) [27,28,29]. Indications for TLE and types of periprocedural complications were defined according to the 2017 HRS Expert Consensus Statement on Cardiovascular Implantable Electronic Device Lead Management and Extraction [28].

All procedures were performed using non-powered mechanical systems such as polypropylene Byrd dilator sheaths (Cook^®^ Medical, Bloomington, IN, USA), mainly via the implant vein. If technical difficulties arose, alternative venous approaches and/or additional tools such as Evolution (Cook^®^ Medical, USA), TightRail (Spectranetics, now Philips Healthcare, Andover, MA, USA), lassos and basket catheters were used. Excimer laser sheaths were not used.

All extraction procedures were performed following different organizational models spanning 15 years of experience. In the initial era of lead extraction, the procedures were performed in the electrophysiology laboratory using intravenous analgesia/sedation; then the recommended safety precautions were observed to perform more complex and risky procedures in the operating theater, and finally in the hybrid room under general anesthesia. The core extraction team has consisted of the same very experienced TLE operator and a dedicated cardiac surgeon with an experienced echocardiographist over the last 6 years.

### 2.4. Transthoracic (TTE) and Transesophageal (TEE) Echocardiography before and after TLE

Initially, only TTE was mandatorily used to aid in pacemaker lead extraction, whereas TEE has been a standard tool over the last 6 years. TTE and TEE in our series were performed using Philips iE33 or GE Vivid S 70 machines equipped with X7-2t Live 3D or 6VT-D probes. All recordings were archived. Echocardiographic images were obtained before TLE, during the extraction procedure (continuous TEE monitoring) and after TLE to assess additional masses on the leads: scar tissue, vegetations, adhesions to the veins, cardiac walls and lead-to-lead binding as well as residual vegetation and scar tissue remnants (“ghosts”) after TLE.

### 2.5. Statistical Analysis 

Statistics and Study Groups

According to the study protocol, the patients were divided into five groups depending on the presence and severity of venous stenosis: group 1—no stenosis, group 2—mild stenosis, group 3—moderate stenosis, group 4—severe stenosis and group 5—total venous occlusion. The Shapiro–Wilk test showed that most continuous variables were normally distributed. For uniformity, all continuous variables are presented as the mean ± standard deviation. The categorical variables are presented as number and percentage. In the first step, the Kruskal–Wallis ANOVA test was used to determine whether there were statistically significant differences between groups. Next, the variables achieving *p* < 0.1 were compared using the nonparametric Chi^2^ test with Yates correction (dichotomous data) or the unpaired Mann–Whitney U test (continuous data), as appropriate. Comparisons were made between groups 1 combined with 2 (no or mild LRVSO) vs. groups 4 combined with 5 (advanced LRVSO). Group 3 (borderline stenosis) was excluded from the second step of the comparative analysis to clearly differentiate patients without or with low grade LRVSO from patients with advanced LRVSO. Thus, the variables included in the regression analysis were selected to determine LVRSO risk factors. Univariate and multivariable logistic regression was used to determine which parameters influenced the severity of venous stenosis. The variables achieving *p* < 0.1 in the unpaired Mann–Whitney U test (continuous data) or Chi^2^ test with Yates correction (dichotomous data) were included in the regression analysis. 

Of the derivative variables (highly correlated), only one of them was included in the multivariate analysis. This especially applies to the number and age of leads. 

From the parameters determining the number of leads in the heart, raw data—the number of active and inactive leads were included in the multivariate analysis. 

The presence of the CRTD system was not included in the multivariate analysis because the presence of the ICD electrode in the studied population did not contribute statistically to the occurrence of LRVSO. The electrode’s influence on LVRSO does not result from the type of leads but from their number (similarly to the CRT-P system) and is represented in the analysis by the variable: “number of the leads in the system before TLE”.

There was one exception. The Kruskal–Wallis ANOVA test, Mann–Whitney U test, and univariate and multivariable regression analyses showed that the CHA2DS2-VASc score predicted the severity of venous stenosis. Therefore, although the frequency of most components of the score (stroke, vascular atherosclerotic disease, diabetes, heart failure) did not differ between groups, they were included into univariate regression. Of the remaining parameters, univariate analysis showed that female sex had a protective predictive value, while older age at first implantation and arterial hypertension were the risk factors for stenosis/occlusion in the venous system. 

Finally, three multivariable models were built. 

Model 1 included the classic CHA2DS2-VASc score points, RV diastolic diameter, presence of atrial fibrillation, number of leads in the system, number of abandoned leads, CS lead presence before TLE, upgrading or additional lead implantation before TLE, cumulative extracted leads dwell time before TLE, connective tissue surrounding the lead and strong connective tissue scar connection of the lead with RA wall. 

Model 2 included a combined parameter (sum of point values for the age at the first implantation: <65 years—0 points, 65–74 years—1point, and ≥75 years—2 points, presence of arterial hypertension—1 point, and male gender—1 point), RV diastolic diameter, presence of atrial fibrillation, number of the leads in the system, number of abandoned leads, CS lead presence before TLE, upgrading or additional lead implantation before TLE, cumulative dwell time of extracted leads before TLE, connective tissue surrounding the lead and strong connective tissue scar connection of the lead with RA walls. 

Model 3 comprised the individual components of the combined measure, i.e., patient’s age at the first implantation, male gender, presence of arterial hypertension, RV diastolic diameter, presence of atrial fibrillation, number of the leads in the system, number of abandoned leads, CS lead presence before TLE, upgrading or additional lead implantation before TLE, cumulative dwell time of extracted lead before TLE, connective tissue surrounding the lead and strong connective tissue scar connection of the lead with RA wall. 

When the original CHA2DS2-VASc score and the combined measure were analyzed, their individual components were not included in the analysis. 

A *p*-value less than 0.05 was considered as statistically significant.

Statistical analysis was performed using Statistica version 13.3 (TIBCO Software Inc., Palo Alto, CA, USA).

### 2.6. Approval of the Bioethics Committee

All patients gave their informed written consent to undergo TLE and use anonymous data from their medical records, approved by the Bioethics Committee at the Regional Chamber of Physicians in Lublin no. 288/2018/KB/VII. The study was carried out in accordance with the ethical standards of the 1964 Declaration of Helsinki.

## 3. Results

Analysis was performed in a total of 2909 patients (39.44% of females) with an average age of 66.90 years. Ischemic heart disease was the leading underlying heart disease (57.61%). Left ventricular ejection fraction (LVEF) was 48.89% on average, and the Charlson comorbidity index was 4.77. Indications for TLE included systemic infection with or without pocket infection (20.59%) and local (pocket) infection (8.70%). 

Among non-infectious indications, there was lead failure (replacement) (57.74%), change of pacing mode/upgrading, downgrading (6.05%) and other (12.86%). Most patients (69.20%) had a pacemaker (PM), 22.93% of patients had an implantable cardioverter-defibrillator (ICD), and only 7.80% received cardiac resynchronization therapy (CRT-D). Implant duration expressed as the mean dwell time of the oldest lead per patient before TLE was 101.5 months, and the cumulative dwell time of the leads before TLE was 15.31 years. 

The rate of major and minor complication was 2.10% and 5.98%, respectively. 

Major complications in our analyzed group of 2909 patients were: hemopericardium needing rescue sternotomy in 28 (1.0%) patients, hemopericardium needing pericardiocentesis 8 (0.3%), hemothorax treated with chest tube insertion in 2 (0.1%), hemothorax needing thoracotomy in 3 (0.1%), brain embolus (with full rehabilitation) in 1 (0.03%), acute heart failure in 2 (0.1%), pulmonary embolus needing open chest surgery in 1 (0.03%), severe tricuspid valve damage 13 (0.4%), mixed hemopericardium plus tricuspid valve damage in 2 (0.1%), hemopericardium needing pericardiocentesis plus tricuspid valve damage 1 (0.03%) [28,29].

Minor complications in our 2909 group were: tricuspid valve damage not needing cardiac surgery in 99 (3.4%) patients, epicardial fluid appearance needing observation only in 33 (1.1%), haemothorax appearance needing only observation in 20 (0.7%), necessary blood transfusion in 20 (0.7%), auxillary vein thrombosis treated conservatively in 12 (0.4%), lead fragment lost without consequences in 1 (0.03%), pneumothorax needing chest tube in 7 (0.2%), pulmonary embolism treated conservatively in 9 (0.3%), mixed in 8 (0.3%) [28,29].

Clinical success, defined as removal of targeted leads and material even with retention of a small portion of a lead that does not negatively impact the outcome goals of the procedure (tip or a small part <4 cm of the lead when the residual part does not increase the risk of secondary complications and with absence of any permanently disabling complication or procedure-related death) was achieved in 98.0% [27,28,29].

Procedural success, defined as removal of all targeted leads and material with the absence of any permanently disabling complication or procedure-related death was achieved in 95.4% of patients [27,28,29].

Patient Groups

For the purposes of analysis, the study population was retrospectively divided into five groups according to venogram results, namely group 1—no stenosis (499 patients), group 2—mild stenosis (574 pts), group 3—moderate stenosis (605 pts), group 4—severe stenosis (581 pts) and group 5—total occlusion (650 pts). Only maximal venous narrowing was considered as a criterion in patient selection.

Table 1, Table 2 and Table 3 compare patient groups with varying degrees of LRVSO in order to establish potential patient-, system- and previous procedure-related risk factors for the build-up of scar tissue being the cause of LRVSO. Table 4 presents the results of univariate and multivariable linear regression analysis of factors potentially influencing the occurrence of LRVSO.

The degree of LRVSO was greater in men, in patients with their first CIED implantation at an older age, with a higher Charlson comorbidity index, more points on the CHA2DS2-VASc scale, higher value of the combined measure (male gender, presence of hypertension, age at first CIED implantation) and those with a higher concentration of creatinine (the borderline of statistical significance). Total venous occlusion was also most common in patients with mechanical valves. Permanent atrial fibrillation, right ventricular enlargement and female sex were shown to have a protective influence on the occurrence of LRVSO (Table 1).

Other patient-related risk factors: baseline heart disease, functional NYHA III and IV class, left ventricular ejection fraction (LVEF), diabetes, long-term anticoagulation and long-term antiplatelet treatment did not show any relationship with the severity of lead-related venous stenosis/occlusion (Table 1).

Analysis of system- and procedure-related risk factors demonstrated that the degree of LRVSO was greater in patients with CRT-D devices, coronary sinus (CS) leads, multiple leads, abandoned leads, especially multiple, and in patients with upgrading or additional lead implantation. Patients with greater degrees of LRVSO were characterized by longer cumulative dwell time of the leads, although the mean implant duration did not differ between the groups (Table 2).

Patients with single-lead devices of the PM type (AAI, VVI, VDD) were less likely to have advanced LRVSO (Table 2). Therapeutic indication for TLE were all class 1 indications (infection, threatening lead, necessity to rebuild venous approach for new lead implantation). Prophylactic indications were all class 2b indications (when the lead may be potentially threatening in the future; they include extractions for unnecessary lead abandonment prevention.

Analysis of relationships between echocardiographic findings and the severity of lead-related venous stenosis/occlusion showed that LVEF and the condition of the tricuspid valve were not related to the degree of LRVSO (Table 3).

As in the case of lead-related infective endocarditis (LRIE), significantly more vegetations were found in patients with advanced LRVSO. Strong adhesion between the lead and right atrial (RA) wall was less common in the LRVSO group, but, on the other hand, multiple ghosts after TLE were significantly more frequent in patients with severe stenosis/occlusion (Table 3).

Univariate regression analysis showed that, of the clinical data, patient age at first implantation (OR = 1.009, *p* < 0.001), gender—male (OR = 1.230; *p* = 0.013), —female (OR = 0.813; *p* = 0.013), right ventricular diastolic diameter (OR = 0.979; *p* = 0.003), atrial fibrillation (OR = 0.751; *p* = 0.004), arterial hypertension (OR = 1.255; *p* = 0.012) and the CHA_2_DS_2_-VASc score (OR = 1.083; *p* < 0.001) were associated with the presence of venous stenosis/occlusion.

Of the CIED-related data, the number of leads in the system (OR = 1.591; *p* < 0.001), the number of abandoned leads (OR = 1.704; *p* < 0.001) (the overall number of leads per patient; OR = 1.664; *p* < 0.001), the presence of more complex systems with coronary sinus leads (OR = 1.401; *p* < 0.001), system upgrading (OR = 1.569; *p* < 0.001) and the cumulative dwell time of extracted leads were related to the presence of venous stenosis/occlusion. Interestingly, the CHA2DS2-VASc score predicted the occurrence of venous stenosis/occlusion, although four of its components (stroke, history of vascular disease history, diabetes, and heart failure) were not related to LRVSC when using ANOVA, Mann–Whitney U test and regression analysis. Therefore, the combined measure was created to include only three items: gender (male), age and arterial hypertension, assigning them the same number of points as on the CHA2DS2-VASc scale. A one-point difference in the combined measure was associated with an increase in LVRSO by 10.4% (OR = 1.104; *p* < 0.001) (Table 4).

Taking into account the ambiguous role of the individual components of the CHA2DS2-VASc score to predict LRVSC, three models of multivariate analysis were created. Model 1, which included the classic CHA2DS2-VASc score, showed the prognostic value of RV dimension (OR = 0.981; *p* = 0.022), atrial fibrillation (OR = 0.668; *p* < 0.001), the number of points on the CHA2DS2-VASc scale (OR = 0,009), and the number of system and abandoned leads (OR = 1.385; *p* < 0.001, OR = 2.008; *p* < 0.001). The presence of one coronary sinus lead (OR = 1.189; *p* = 0.052) had borderline significance. The results of Model 2 were similar, although the predictive value of the combined measure was higher compared to the CHA2DS2-VASc score (OR = 1.220; *p* < 0.001), and the presence of coronary sinus leads was statistically significant (OR = 1.207; *p* = 0.033) (Table 4).

Model 3 showed the predictive value of patient age at first CIED implantation (OR = 1.008; *p* = 0.021), gender (OR for men = 1.349; *p* = 0.003), right ventricular diastolic diameter (OR = 0.978; *p* = 0.006), atrial fibrillation (OR = 0.666; *p* < 0.001), the number of leads in the system and abandoned leads (OR = 1.345; *p* = 0.003, OR = 1.965; *p* < 0.001, respectively)), whereas the presence of coronary sinus leads had borderline significance (OR = 1.184; *p* = 0.056) (Table 4).

## 4. Discussion

Obstruction of the large veins of the thorax is a well-known complication after the implantation of a permanent transvenous pacemaker. The incidence of venous obstruction reaches 30–45%, with an average complete occlusion rate of 12.2% and a symptomatic occlusion rate of 1–3% [1,2,3,4,5,6,7,8,9,10,11,12,13,14,15,16,17,18,19,20,21,22]. There is a large number of studies that describe the risk of LRVSO [1,2,3,4,5,6,7,9,10,11,13,14,15,16,17,18,19,20], but they were performed in relatively small cohorts of patients and only some of them analyzed the system-related risk factors for venous obstruction [1,2,3,4,5,6,7,10,11,13,14,15,16,18,19,20]. However, it is still reasonable to expect that the identification of modifiable patient-related, system-/lead-related and lead management-related risk factors can help reduce the incidence and severity of LRVSO.

Considering potential patient-related risk factors, several studies demonstrated that low LVEF increased [1,4,12] or had no influence [7], AF increased [4,14] or had no effect [1,7,11], and gender had no influence (all authors agree) [1,2,4,5,6,7,8,9,11,13,15,16,18,19] on the risk of LRVSO. According to two authors permanent anticoagulation/antiplatelet treatment reduces the risk of LRVSO [5,11] but most investigators state that there is no influence [2,6,10,18], one author found out that diabetes reduced the risk of LRVSO [6] but not others [7,11,18].

Contrary to the previous report [30], the present study did not demonstrate the protective role of the CHA2DS2-VASc score in preventing LRVSO. Multivariate analysis in our study showed that a 1-point difference in the CHA2DS2-VASc score increased the likelihood of severe stenosis or lead-related venous obstruction by 7.8%. Apart from patient age and gender, other clinical variables included in the scale had no prognostic value.

Additionally, in the present cohort of 2909 patients, the incidence of LRVSO was unrelated to baseline heart disease as the cause of CIED implantation, diabetes, and chronic antiplatelet and anticoagulation therapy.

Considering potential system-related risk factors for LRVSO, there is evidence that the number of leads (lead burden) either increases the risk of LRVSO [2,5,11,14,18] or has no effect [1,3,6,7,10,13,15,16,20]. Similarly, some investigators consider lead caliber as a risk factor for LRVSO [9,11,13,14,17,18] but not others [1,2,3,4,5,6,7,15,16,19,20]. As regards implant duration all investigators agree that it has no significant influence on the risk of LRVSO [1,11,15,16,19].

The natural history of LRVSO and its progression remain unclear. Lead-related endothelial injury may cause an inflammatory response of the vessel wall with subsequent thrombosis and scarring [23]. In our opinion, the factors that predispose to LRVSO are male gender, CIED implantation in older age, multi-lead systems, especially with left ventricular leads, and the presence of abandoned leads. A larger diameter of the right ventricle at end-diastole and the presence of atrial fibrillation had an opposite i.e., protective effect.

Multiple lead implantation or additional lead implantation with abandonment of inactive leads induces further damage to the endothelium. The role of thrombosis in delayed (months) or late (years) LRVSO is less clear [23]. The inflammatory response of the vessel wall probably incites the formation of scar tissue similar to the scar binding the lead to the vessel and heart structures detected around the extracted leads and on TEE (multiple ghosts) [31,32,33,34,35]. In this aspect, permanent anticoagulation may reduce the risk of early thrombosis [5,11] but it has no impact on the subsequent formation of the connective tissue. The above-cited investigators performed their examinations at different intervals after first system implantation or during planned follow-up (6–18 months) [1,3,4,5,18,20,21], later (41, 45, 46 months) [7,19,22], during any next CIED procedure [9,11,12,15], or only before TLE [2,6,13,16]. Limited numbers of patients (100–150 pts [3,4,5,6,7,8,9,10,11,12] and 30–89 pts [13,14,15,16,17,18,19,20,21,22]) and varying intervals after implantation to venography make it difficult to draw reliable conclusions from the literature data.

The present study demonstrated that general health status and patient-related risk factors for major TLE complications (baseline heart disease, functional NYHA class, LVEF, co-existing diseases) had no impact on the risk of LRVSO, similarly to long-term anticoagulation and long-term antiplatelet treatment. The current study also showed that the prognostic value of risk assessment using the CHA_2_DS_2_-VASc score requires further analysis, due to the predominant role of other combined risk factors: the number of leads in the system and abandoned leads, the presence of coronary sinus leads, age at first CIED implantation, male gender, right ventricular diastolic diameter, and the presence of atrial fibrillation.

The present findings indicate that the essential cause of LRVSO is mechanical irritation of the venous wall by the implanted leads. It is related to the lead number, the force of adhesion to the venous wall, and lead mobility. The force of adhesion to the venous wall and lead mobility may be lower in patients with right ventricular dilatation. In turn, in patients with atrial fibrillation, lead movement caused by atrial mechanical activity is suppressed. A more frequent occurrence of LRVSO in males is probably due to anatomical conditions—the pocket most often lies on the pectoralis major muscle, and the greater physical activity of men translates into a mechanical effect on the implanted leads.

It is worth noting that lead abandonment is the only operator-related factor predisposing to LRVSO. Severe lead-related venous stenosis or total venous occlusion is twice as high in patients with abandoned leads.

## 5. Conclusions

The present findings indicate that the main cause of LRVSO is the mechanical impact of the implanted lead on the vein wall with subsequent development of scar tissue, therefore, the system-/procedure-related risk factors show a more significant correlation with the severity of LRVSO. The most important risk factors for LRVSO are lead burden, CS leads and abandoned leads. Among patient-related factors, only male gender and patient age at first CIED implantation were significant risk factors for LRVSO. The role of the thrombotic component probably becomes less important long after implantation, therefore the CH_2_DS_2_-VASc score used to predict the maximum degree of LRVSO, especially in patients with long implant duration, may not be reliable.

## 6. Study Limitations

This study has some limitations. Routine venography before TLE was performed in all patients except those with contraindications, mainly renal failure. That was the reason why this interesting patient subpopulation was excluded from the study. The database was prospectively integrated, but analysis was performed retrospectively. For the purpose of this study, the population of patients was divided into groups according to maximal venous narrowing without taking into account the site of narrowing/occlusion and the length of venous stenosis/occlusion. Therefore, the present analysis of venograms includes maximal venous narrowing but not the volume of the phenomenon (the number of vessels affected). The classification of patients we used in the study not only enabled comparison of our results with the findings of other investigators, but also maximal venous narrowing was considered a practical marker for predicting reuse of veins for implantation of a new lead/catheter.

## Figures and Tables

**Table 1:** Potential patient-related risk factors for lead-related venous stenosis/occlusion.

	No Stenosis 1	Mild Stenosis 2	Moderate Stenosis 3	Severe Stenosis 4	Total Occlusion 5	ANOVA Kruskal-Wallis Test (1–5) P	Mann-Whitney U/Ch^2^ Tests (1–2) vs. (4–5)
Number of Patients	*N* = 499	*N* = 574	*N* = 605	*N* = 581	*N* = 650		
	Mean ± SD *n* (%)	Mean ± SD *n* (%)	Mean ± SD *n* (%)	Mean ± SD *n* (%)	Mean ± SD *n* (%)		
Potential patient-related risk factors							
Patient age at first implantation (years)	56.42 ±16.13	58.12 ±15.19	58.12 ±15.36	60.76 ±14.41	58.81 ±16.77	<0.001	<0.001
Male gender	325 (65.13)	324 (56.44)	324 (53.55)	342 (58.86)	447 (68.77)	<0.001	0.082
Baseline heart disease: ischemic heart disease	278 (55.71)	329 (57.31)	353 (58.34)	361 (62.13)	355 (54.62)	0.112	
Baseline heart disease: cardiomyopathy	69 (13.83)	72 (12.54)	80 (13.22)	74 (12.74)	85 (13.08)	0.675	
Baseline heart disease: valvular heart disease	14 (2.806)	17 (2.962)	8 (1.322)	11 (1.892)	18 (2.796)	0.169	
Baseline heart disease: congenital, channelopathies, neurocardiogenic	138 (27.66)	156 (27.17)	163 (26.94)	135 (23.24)	192 (29.54)	0.229	
NYHA class III & IV	67 (13.42)	96 (16.72)	71 (11.73)	196 (33.74)	99 (15.23)	0.523	
LVEF (%)	49.52 ±15.75	48.86 ±15.41	49.46 ±15.22	48.34 ±14.76	48.39 ±15.00	0.386	
PASP (mm Hg)	30.82 ±13.58	29.98 ±13.15	30.15 ±12.71	31.94 ±13.51	30.67 ±13.48	0.137	
RV diameter (mm)	32.09 ±6.676	31.04 ±5.623	30.69 ±5.888	30.91 ±5.952	30.62 ±5.618	<0.001	<0.001
AF permanent	148 (29.66)	138 (24.04)	128 (21.15)	123 (21.17)	138 (21.23)	0.003	0.003
Arterial hypertension	262 (52.51)	339 (59.06)	365 (60.33)	365 (62.82)	389 (59.85)	0.011	0.012
Congestive heart failure	104 (20.94)	110 (19.16)	114 (18.84)	106 (18.24)	110 (16.92)	0.515	
Prior stroke or TIA or thromboembolism	38	49	47	54	54		
Vascular atherosclerotic disease	221	247	260	268	289		
Diabetes (any)	103 (20.64)	111 (19.33)	122 (20.16)	132 (22.72)	124 (19.08)	0.545	
Renal failure, mild	75 (15.03)	98 (17.07)	102 (16.86)	109 (18.76)	132 (20.310	0.165	
Renal failure, severe	11 (2.204)	18 (3.136)	17 (2.810)	7 (1.205)	19 (2.923)	0.924	
Creatinine level (mg/dL)	1.145 ±0.604	1.138 ±0.493)	1.137 ±1.155	1.155 ±0.628	1.225 ±0.787	0.056	0.066
BMI (kg/m^2^)	28.22 ±4.377	28.31 ±8.234	27.91 ±4.374	28.19 ±4.512	27.55 ±4.359	0.056	0.592
Valve implant	46 (9.128)	37 (6.446)	36 (5.950)	31 (5.336)	65 (10.00)	0.088	0.983
Mechanical valve	29 (5.812)	20 (3.484)	34 (5.620)	15 (2.582)	48 (7.385)	0.006	0.606
Long-term anticoagulation	220 (44.08)	229 (39.89)	227 (37.52)	221 (38.04)	277 (42.62)	0.126	
Long-term antiplatelet treatment	210 (42.08)	266 (46.34)	272 (44.95)	284 (48.88)	279 (42.92)	0.174	
Charlson comorbidity index (points)	4.543 ±3.789	4.702 ±3.380)	4.688 ±3.589	5.086 ±3.629	4.728 ±3.550	0.038	0.016
CHA_2_DS_2_–VASc score (points)	2.812 ±1.745	3.028 ±1.666)	3.041 ±1.639	3.374 ±1.621	3.023 ±1.025	0.010	<0.001
Combined parameter (male gender—1 point, <65 years—0 points, 65–74 years—1 point, ≥75 years—2 points, arterial hypertension—1 point) (points)	1.643 ±1.017	1.650 ±1.020	1.636 ±1.041	1.813 ±1.043	1.840 ±1.037	<0.001	<0.001
HAS-BLED score (points)	1.317 ±1.037	1.448 ±1.045	1.435 ±1.032	1.575 ±1.007	1.500 ±1.025	0.439	

TLE—transvenous lead extraction, NYHA—New York Heart Association functional class, LVEF—left ventricular ejection fraction, PASP—pulmonary artery systolic pressure, RV—right ventricle, AF—atrial fibrillation, BMI—body mass index, CHA_2_DS_2_–VASc—score to predict the risk of thromboembolic complications, HAS-BLED—score to predict bleeding complications, ICD-V—single-chamber implantable cardioverter-defibrillator, ICD-D—dual-chamber implantable cardioverter-defibrillator, CRTD—implantable cardioverter-defibrillator with resynchronization function, CVS—coronary sinus.

**Table 2:** Potential indication-related, system-related and history of pacing-related risk factors for lead-related venous stenosis/occlusion.

	No Stenosis 1	Mild Stenosis 2	Moderate Stenosis 3	Severe Stenosis 4	Total Occlusion 5	ANOVA Kruskal-Wallis Test (1–5) P	Mann-Whitney U/Ch^2^ Tests (1–2) vs. (4–5)
Number of Patients	*N* = 499	*N* = 574	*N* = 605	*N* = 581	*N* = 650		
	Mean ± SD *n* (%)	Mean ± SD *n* (%)	Mean ± SD *n* (%)	Mean ± SD *n* (%)	Mean ± SD *n* (%)		
TLE indications							
LRIE certain with or without pocket infection	65 (13.03)	69 (12.02)	81 (13.39)	93 (16.01)	133 (20.46)	<0.001	<0.001
LRIE probable with or without pocket infection	24 (4.810)	26 (4.530)	34 (5.620)	44 (7.573)	42 (6.462)	0.073	0.023
Local (pocket) infection (only)	43 (8.617)	53 (9.233)	52 (8.595)	34 (5.851)	62 (9.538)	0.145	
Infection (all)	132 (26.45)	148 (25.78)	167 (27.60)	171 (29.43)	237 (36.46)	<0.001	<0.001
Non-infectious indications: prophylactic	16 (3.206)	21 (3.659)	29 (4.793)	24 (4.131)	10 (1.538)	0.037	0.407
Non-infectious indications: therapeutic	351 (70.34)	405 (70.56)	409 (67.60)	386 (66.44)	403 (62.00)	0.018	0.014
**System and History of Pacing**							
Device type—PM AAI, VVI, VDD (single lead)	120 (24.05)	134 (23.35)	110 (18.18)	102 (17.56)	120 (18.46)	0.012	<0.001
Device type—PM DDD (dual lead)	220 (44.09)	262 (45.65)	292 (48.26)	264 (45.44)	321 (49.39)	0.420	
Device type—CRT-P (three leads)	10 (2.004)	6 (1.045)	14 (2.314)	18 (3.098)	20 (3.077)	0.017	0.017
Device type—ICD-V. ICD-D	126 (25.25)	127 (22.13)	144 (23.80)	141 (24.27)	127 (19.54)	0.142	
Device type—CRT-D	21 (4.208)	44 (7.666)	44 (7.237)	56 (9.639)	62 (9.54)	0.006	0.002
Number of leads in the system before TLE (*n*)	1.653 ±0.589	1.777 ±0.652	1.846 ±0.610	1.900 ±0.638	1.902 ±0.654	<0.001	<0.001
Abandoned leads before TLE	36 (7.214)	46 (8.014)	41 (6.778)	63 (10.83)	112 (17.23)	<0.001	<0.001
Number of abandoned leads before TLE	0.078 ±0.290	0.112 ±0.411	0.086 ±0.344	0.145 ±0.451	0.231 ±0.557	<0.001	<0.001
Multiple abandoned leads before TLE	3 (0.601)	16 (2.787)	10 (1.653)	19 (3.270)	33 (5.077)	<0.001	<0.001
Number of leads in the heart before TLE (sum of leads in the system and abandoned leads)	1.732 ±0.640	1.883 ±0.720	1.926 ±0.653	2.038 ±0.728	2.126 ±0.836	<0.001	<0.001
≥4 leads in heart before TLE	1 (0.200)	15 (2.613)	8 (1.322)	16 (2.754)	44 (6.769)	<0.001	<0.001
≥5 leads in heart before TLE	0 (0.00)	1 (0.174)	0 (0.00)	2 (0.344)	6 (0.923)	0.021	0.072
HV lead before TLE	149 (29.86)	173 (30.14)	189 (31.24)	197 (33.91)	190 (29.23)	0.212	
One single-coil ICD lead before TLE	68 (13.63)	79 (13.76)	68 (11.24)	96 (16.52)	87 (13.39)	0.121	
Dual-coil ICD lead before TLE	79 (15.83)	92 (16.03)	116 (19.17)	96 (16.52)	99 (15.23)	0.384	
Two single-coil ICD leads before TLE	3 (0.601)	3 (0.523)	3 (0.496)	5 (0.861)	2 (0.308)	0.772	
Two dual-coil ICD leads before TLE	0 (0.00)	1 (0.174)	2 (0.331)	0 (0.00)	1 (0.154)	0.522	
Three ICD leads before TLE	0 (0.00)	0 (0.00)	0 (0.00)	0 (0.00)	1 (0.154)	0.486	
CS lead before TLE	47 (9.419)	82 (14.29)	99 (16.36)	124 (21.34)	126 (19.39)	<0.001	<0.001
Leads on the left side of the chest before TLE	473 (94.79)	550 (95.82)	582 (96.20)	550 (94.66)	602 (92.52)	0.241	
Leads on the right side of the chest before TLE	20 (4.008)	10 (1.742)	18 (2.975)	13 (2.238)	16 (2.462)	0.185	
Leads on both sides of the chest before TLE	6 (1.202)	14 (2.439)	5 (0.826)	17 (2.926)	32 (4.923)	<0.001	0.004
Previous TLE before present TLE	23 (4.609)	23 (4.007)	19 (3.140)	27 (4.647)	39 (6.00)	0.200	
History of early CIED intervention	13 (2.605)	28 (4.878)	26 (4.298)	9 (1.549)	26 (4.00)	0.186	
Upgrading or additional lead implantation	26 (5.210)	71 (12.37)	71 (11.74)	83 (14.29)	119 (18.31)	<0.001	<0.001
Upgrading or downgrading with lead abandonment	13 (2.605)	25 (4.355)	28 (4.628)	43 (7.401)	72 (11.08)	<0.001	<0.001
Last CIED procedure excluding pocket repair (months)	47.80 ±36.47	50.06 ±38.63	48.74 ±39.77	46.71 ±35.13	44.42 ±34.75	0.033	0.063
Dwell time of the oldest lead per patient before TLE (months)	97.31 ±74.60	104.3 ±75.05	103.0 ±78.57	94.59 ±70.30	107.1 ±14.33	0.028	0.795
Mean implant duration before TLE (months)	92.04 ±66.67	96.86 ±66.15	95.93 ±70.14	87.55 ±61.93	96.15 ±66.61	0.120	0.220
Cumulative dwell time of extracted leads before TLE (years)	13.52 ±12.16	15.40 ±12.98	15.38 ±12.58	14.75 ±11.97	17.03 ±14.34	<0.001	<0.001

TLE—transvenous lead extraction, LRIE—lead-derived infective endocarditis, NYHA—New York Heart Association functional class, LVEF—left ventricular ejection fraction, PASP—pulmonary artery systolic pressure, RV—right ventricle, AF—atrial fibrillation, BMI—body mass index, PM—pacemaker, AAI—one lead atrial pacemaker, VVI—one lead ventricle pacemaker, VDD—one lead, double chamber pacemaker (atrial sensing, ventricle sensing/pacing), DDD—double leads, double chamber pacemaker, CRTP—three leads cardiac resynchronization therapy pacemaker, ICD-V—single-chamber implantable cardioverter-defibrillator, ICD-D—dual-chamber implantable cardioverter-defibrillator, CRTD—implantable cardioverter-defibrillator with resynchronization function, HV lead—defibrillation lead, CS—coronary sinus, CIED—cardiac implantable electronic device.

**Table 3:** Echocardiographic findings/abnormalities in patients with various degrees of lead-related venous stenosis/occlusion.

	No Stenosis 1	Mild Stenosis 2	Moderate Stenosis 3	Severe Stenosis 4	Total Occlusion 5	ANOVA Kruskal-Wallis Test (1–5) P	Mann-Whitney U/Ch^2^ Tests (1–2) vs. (4–5)
Number of Patients	*N* = 499	*N* = 574	*N* = 605	*N* = 581	*N* = 650		
	Mean ± SD *n* (%)	Mean ± SD *n* (%)	Mean ± SD *n* (%)	Mean ± SD *n* (%)	Mean ± SD *n* (%)		
**ECHO before and after TLE**							
LVEF average	49.52 ±15.75	48.86 ±15.43	49.46 ±15.23	48.34 ±14.76	48.39 ±15.00	0.386	
Preserved LVEF (≥50%)	273 (54.71)	298 (51.92)	335 (55.37)	299 (51.46)	333 (51.23)	0.678	
Mid-range LVEF (40–49%)	74 (14.82)	96 (16.72)	89 (14.71)	103 (17.73)	101 (15.53)	0.238	
Reduced LVEF (≤ 40%)	152 (30.46)	180 (31.36)	181 (29.92)	179 (30.81)	216 (33.23)	0.809	
**Tricuspid regurgitation before TLE**							
Non-significant/small	372 (74.54)	451 (78.57)	497 (82.15)	444 (76.42)	492 (75.69)	0.044	0.749
Significant	80 (16.03)	75 (13.07)	70 (11.57)	91 (15.66)	97 (14.69)	0.120	
Severe	25 (5.01)	22 (3.833)	21 (3.471)	25 (4.303)	28 (4.308)	0.744	
**Any shadows on leads before TLE**							
Any shadows on leads before TLE	212 (42.49)	250 (43.55)	265 (43.80)	260 (44.75)	297 (45.69)	0.230	
Scar tissue surrounding the lead	50 (10.02)	47 (8.188)	55 (9.091)	52 (8.950)	34 (5.231)	0.067	0.082
Blood clot on the lead	19 (3.808)	37 (6.446)	39 (6.446)	39 (6.713)	40 (6.154)	0.278	
Vegetation-like masses	16 (3.206)	22 (3.833)	23 (3.802)	34 (5.852)	26 (4.00)	0.194	
Lead thickening	82 (16.43)	79 (13.76)	97 (16.03)	80 (13.77)	95 (14.61)	0.513	
True vegetation	61 (12.22)	66 (1.498)	83 (13.72)	90 (15.49)	124 (19.08)	*p* < 0.001	<0.001
Strong adhesion between the lead and heart structures (any)	60 (12.02)	78 (13.59)	68 (11.24)	55 (9.47)	65 (10.00)	0.119	
Strong adhesion between the lead and tricuspid apparatus	20 (4.01)	25 (4.355)	29 (4.793)	19 (3.270)	32 (4.923)	0.515	
Strong adhesion between the lead and VCS	12 (2.41)	21 (3.659)	26 (4.298)	15 (2.582)	22 (3.385)	0.390	
Strong adhesion between the lead and RA wall	25 (5.01)	23 (4.007)	15 (2.479)	20 (3.442)	14 (2.154)	0.051	0.036
Strong adhesion between the lead and RV wall	27 (5.41)	33 (5.749)	35 (5.785)	19 (3.270)	34 (5.231)	0.241	
**Ghosts after TLE**							
Scar tissue (ghosts) after TLE	129 (25.85)	132 (23.00)	164 (27.11)	142 (24.44)	179 (27.54)	0.241	
Length of ghost after TLE	21.90 ±16.10	20.73 ±13.82	20.57 ±16.29	20.82 ±13.12	22.43 ±14.31	0.278	
Width of ghost after TLE	3.895 ±19.24	4.456 ±1.854	3.784 ±1.673	4.044 ±1.641	3.864 ±1.632	0.608	
Single ghost	96 (19.24)	97 (16.90)	125 (20.66)	108 (18.59)	114 (17.54)	0.586	
Multiple ghosts	25 (5.01)	35 (6.10)	39 (6.45)	34 (5.85)	64 (9.86)	0.020	0.031

ECHO—echocardiographic imagination, TLE—transvenous lead extraction, LVEF—left ventricle ejection fraction.

**Table 4:** Univariate and multivariable analysis of risk factors for lead-related venous stenosis/occlusion.

	Univariate Analysis			Multivariable Model 1			Multivariable Model 2			Multivariable Model 3		
	OR	95%CI	*P*	OR	95%CI	*P*	OR	95%CI	*P*	OR	95%CI	*P*
Patient age at first system implantation (by one year)	1.009	1.004–1.015	0.000							1.008	1.001–1.016	0.021
Male gender	1.230	1.045–1.448	0.013							1.349	1.110–1.638	0.003
RV diameter (mm)	0.979	0.965–0.993	0.003	0.981	0.966–0.997	0.022	0.980	0.964–0.966	0.013	0.978	0.962–0.994	0.006
AF permanent	0.751	0.619–0.911	0.004	0.668	0.528–0.845	0.001	0.669	0.530–0.845	0.001	0.666	0.526–0.842	0.001
Creatinine (by one mg/dL)	1.102	0.974–1.248	0.123									
Charlson comorbidity index	1.019	0.996–1.042	0.100									
CHA_2_DS_2_–VASc score (points)	1.083	1.031–1.137	0.001	1.078	1.019–1.140	0.009						
Congestive heart failure	0.850	0.689–1.048	0.128									
Arterial hypertension	1.255	1.052–1.479	0.012							1.113	0.913–1.358	0.290
Diabetes t. 2	1.047	0.856–1.281	0.652									
History of Stroke/TIA/Thromboembolism	1.085	0.808–1.457	0.588									
History of vascular disease	1.060	0.898–11.25	0.491									
Patient age at first system implantation (>65), (65–74), (≥75) (0—1—2 points)	1.246	1.124–1.380	0.001									
Female gender (yes/no)	0.813	0.691–0.957	0.013									
Combined parameter (age at first implantation, arterial hypertension, male gender) (points)	1.104	1.053–1.154	0.001				1.220	1.108–1.344	0.000			
Valve implant (yes/no)	0.990	0.775–1.263	0.932									
Mechanical implant (yes/no)	1.084	0.727–1.616	0.692									
Systemic infection (LRIE) (yes/no)	1.674	1.363–2.056	0.000									
Device type—PM AAI, VVI, VDD (single lead)	0.714	0.584–0.874	0.001									
Device type—CRT-P (yes/no)	2,095	1,161–3,780	0,014									
Device type—CRT-D (yes/no)	1.636	1.195–2.241	0.002									
Number of leads in the system (by one)	1.591	1.392–1.819	0.000	1.385	1.141–1.682	0.001	1.349	1.111–1.638	0.002	1.345	1.104–1.638	0.003
Abandoned leads before TLE (yes/no)	2.060	1.559–2.720	0.000									
Number of abandoned leads before TLE	1.704	1.387–2.095	0.000	2.008	1.499–2.690	0.000	1.967	1.464–2.641	0.000	1.965	1.462–2.642	0.000
Multiple abandoned leads before TLE (yes/no)	2.533	1.492–4.303	0.001									
Number of leads in the heart before TLE	1.664	1.479–1.871	0.000									
≥4 leads in heart before TLE (yes/no)	6.982	0.871–55.98	0.067									
≥5 leads in heart before TLE (yes/no)	3.472	1.955–6.164	0.000									
CS lead before TLE (yes/no)	1.401	1.234–1.590	0.000	1.189	0.999–1.415	0.052	1.207	1.015–1.436	0.033	1.184	0.996–1.409	0.056
Upgrading or additional lead implantation (yes/no)	1.569	1.231–1.999	0.000	1.011	0.744–1.373	0.944	1.022	0.745–1.401	0.893	1.013	0.748–1.372	0.935
Upgrading or downgrading with lead abandonment	2.846	1.955–4.144	0.000									
Last CIED procedure excluding pocket repair (months)	0.961	0.936–0.986	0.003									
Dwell time of the oldest lead per patient before TLE (months)	1.003	0.990–1.016	0.702									
Cumulative dwell time of extracted leads before TLE (years)	1.009	1.002–1.015	0.009	0.995	0.986–1.004	0.280	0.998	0.989–1.007	0.705	0.999	0.990–1.009	0.887
Scar tissue surrounding the lead	0.764	0.564–1.034	0.081	0.820	0.595–1.130	0.224	0.827	0.602–1.135	0.239	0.826	0.601–1.136	0.238
Vegetation (yes/no)	1.610	1.268–2.043	0.000									
Strong adhesion between the lead and RA wall	0.606	0.387–0.949	0.029	0.659	0.410–1.060	0.085	0.701	0.438–1.124	0.140	0.714	0.445–1.147	0.163

## Data Availability

Readers can access the data supporting the conclusions of the study at www.usuwanieelektrod.pl (accessed on 2 November 2021).

## Abbreviations

AnV	innominate (brachiocephalic) vein
AxV	axillary vein
CHA2DS2-VASc	clinical prediction rules for estimating the risk of stroke in people with non-rheumatic AF
CIED	cardiac implantable electronic device
CRT	cardiac resynchronization therapy
EF	LV ejection fraction
FU	follow-up
ICD	implantable cardioverter-defibrillator
IVC	inferior vena cava
LR	lead-related
LRVSO	lead-related venous stenosis/occlusion
LV	left ventricle
LVEF	left ventricular ejection fraction
NYHA	The New York Heart Association (functional class)
PASP	pulmonary artery systolic pressure
Pts	patients
PM	pacemaker
RA	right atrium
RV	right ventricle
TEE	transesophageal echocardiography
TLE	transvenous lead extraction
ScV	subclavian vein
SVC	superior vena cava
TV	tricuspid valve
VSO	venous stenosis/occlusion
